# Contributions of Demographics, Language Learning Experience, and Cognitive Control to Chinese Reading Comprehension

**DOI:** 10.3389/fpsyg.2021.770579

**Published:** 2021-11-25

**Authors:** Zhilong Xie, Wei Wang, Xiaying Chu, Qing Qiu, Fangfang Yuan, Jinwen Huang, Meijing Chen

**Affiliations:** ^1^Foreign Languages College, Jiangxi Normal University, Nanchang, China; ^2^School of Intercultural Studies, Jiangxi Normal University, Nanchang, China

**Keywords:** demographics, L2 learning experience, cognitive control, Chinese reading comprehension, Chinese as a foreign/second language

## Abstract

The study investigates whether learners’ demographics (e.g., age, education, and intelligence-IQ), language learning experience, and cognitive control predict Chinese (L2) reading comprehension in young adults. Thirty-four international students who studied mandarin Chinese in mainland China (10 females, 24 males) from Bangladesh, Burundi, Congo, Madagascar, Nigeria, Rwanda, South Africa, and Zimbabwe were tested on a series of measures including demographic questionnaires, IQ test, two cognitive control tasks [Flanker Task measuring inhibition and Wisconsin Card Sorting Test (WCST) measuring mental set shifting], and a Chinese reading comprehension test (HSK level 4). The results of correlation analyses showed that education, L2 learning history, L2 proficiency, and previous category errors of the WCST were significantly correlated with Chinese reading comprehension. Further multiple regression analyses indicated that Chinese learning history, IQ, and previous category errors of the WCST significantly predicted Chinese reading comprehension. These findings reveal that aside from IQ and the time spent on L2 learning, the component mental set shifting of cognitive control also predicts reading outcomes, which suggests that cognitive control has a place in reading comprehension models over and above traditional predictors of language learning experience.

## Introduction

Reading comprehension is important not just for text understanding, but for broader learning, success in education and employment. Reading involves complex and interactive processes that require readers to construct a meaningful representation of a text by using background knowledge, linguistic knowledge, and different cognitive skills and abilities (Rayner and Reichle, 2010; Oakhill et al., 2015). The study of reading comprehension has traditionally focused on readers of native or first language. However, the rapid globalization has promoted a higher necessity for L2 reading. Great efforts have been made in investigating L2 reading of alphabetic languages, particularly L2 reading in English (Harrison and Krol, 2007; Qiao et al., 2021). During more recent years, however, scholars have shown interest in L2 reading involving Asian languages, particularly Chinese, as a result of the globalization of Chinese economy (Zhu, 2021). Written Chinese is different from alphabetic languages in terms of many aspects, such as at the orthographic, the phonetic, the morphological, and the syntactic levels, so reading acquisition of Chinese may be a major challenge for those learning Chinese as a second/foreign language (CSL/CFL). However, relevant studies are sparse. The current study thus intends to fill the gap by investigating among CSL/CFL learners how Chinese reading comprehension may be attributed to multiple factors, including learners’ demographic background, linguistic experience, and cognitive control abilities, as those factors have been reported to be in association with reading comprehension.

The reading processes are multidimensional, which may include word-level processes that encode the visual pattern of a word and access its meaning (word reading/identification), sentence-level processes that compute syntactic parsing and semantic integration (syntactic parsing), text-level processes that establish both the local and global organization of the whole text (discourse representation), and cognitive processes that allocate and coordinate different cognitive resources to interpret meanings (Seigneuric and Ehrlich, 2005; Chung et al., 2020). According to the Simple View of Reading (SVR) proposed by Gough and Tunmer (1986), reading equals the product of decoding and comprehension (*R*=*D*×*C*). Decoding ability varies directly with knowledge of the spelling-sound correspondence rules of a language. It is a skill to read isolated words/pseudo words quickly, accurately, and silently, which is fundamental to word recognition. Comprehension is not reading comprehension, but linguistic comprehension. It is the process by which given lexical information, sentences, and discourses are interpreted. Decoding is necessary but not sufficient for reading. Meanwhile, if there is decoding but no comprehension, reading is not taking place. In the current study, we generally define reading comprehension as sentence and passage comprehension, which includes both decoding and comprehension processes.

Most previous studies focused on the relationship between decoding and reading comprehension, particularly in alphabetic languages (e.g., Jenkins et al., 2003; Rasinski et al., 2005; Yovanoff et al., 2005; Klauda and Guthrie, 2008). There are, however, some studies on (non-alphabetic) Chinese reading comprehension that examined character recognition and comprehension (e.g., Joshi et al., 2012; Yeung et al., 2016; Li et al., 2021). For example, Joshi et al. (2012) found that the SVR as incorporated in the componential model of reading (CMR) is also applicable to Chinese other than English. The results of their study revealed that character recognition and listening (linguistic) comprehension significantly predicted Chinese reading comprehension in Chinese children. Yeung et al. (2016) examined the interrelationships between linguistic comprehension skills, decoding and reading comprehension within Chinese children in a longitudinal study, and found that linguistic comprehension and decoding were the main predictors for a significant amount of variance in sentence and passage comprehension. Similarly, Li et al. (2021) examined how Chinese character decoding and context-driven auditory semantic integration contributed to reading comprehension among Chinese middle school students by administering speech-in-noise tests, and found that performance in Chinese character decoding and auditory semantic integration scores with the flattened (but not natural) fundamental frequency contours of spoken sentences significantly predicted reading comprehension.

It is not surprising that higher language proficiency is related to better reading comprehension (e.g., Cutting and Scarborough, 2006; Jiang, 2011; Uccelli et al., 2015). In a longitudinal study by Uchikoshi et al. (2016), for example, the role of oral proficiency on English reading comprehension was examined among 102 English language learners, including both Spanish and Cantonese speakers. The results of multiple regression analyses showed it was not the quantity and variety of words used in the narratives but the ability to produce a coherent oral narrative in either the home language or English that predicted English reading comprehension.

Some studies (although relatively few) also indicate that there is a relationship between learners’ demographics [e.g., age, socioeconomic status (SES), and IQ] and reading comprehension (e.g., Bowey, 1995; Tiu et al., 2003; Kieffer, 2010; Jefferson et al., 2011; Ghabanchi and Rastegar, 2014). For example, a recent study by Cheng and Wu (2017) examined the relationship between SES and reading development among Chinese young children. The results of mediation model showed that SES exerted its effect on sentence reading comprehension through the indirect path *via* the simple mediating effect of morphological awareness and the three-path mediating effect of vocabulary knowledge and morphological awareness.

More recently, some studies have examined the relationship between cognitive factors and reading comprehension (e.g., Chung et al., 2020; Stasenko et al., 2020; Wu et al., 2020). For example, Chung et al. (2011) identified cognitive abilities that might distinguish Hong Kong Chinese adolescents with dyslexia and to assess how these abilities were associated with Chinese word reading, word dictation, and reading comprehension. In their study, a series of literacy and cognitive skills (morphological awareness, visual-orthographic knowledge, rapid naming, and verbal working memory) were tested among 90 junior secondary school students (30 dyslexic, 30 chronological age controls, and 30 reading level controls). The results showed that the dyslexic students were less competent than the control students in all cognitive and literacy measures, and the regression analyses showed that verbal working memory, rapid naming, morphological awareness, and visual-orthographic knowledge were significantly associated with literacy performance. These findings suggest that the cognitive skills have important role in Chinese literacy acquisition.

To sum up, previous studies mainly focused on L1 reading comprehension and found that reading comprehension is related to multiple aspects, such as decoding, syntactic skills, linguistic comprehension, language proficiency, and cognitive control abilities. However, few studies have ever examined Chinese reading comprehension among CSL/CFL learners and few have ever explored those multiple aspects related with Chinese reading comprehension in a single investigation. Since reading processes are complex and many factors may play differential roles simultaneously in reading comprehension, there is a necessity, from various perspectives, to explore the relationship between multiple variables (i.e., demographics, language learning, and cognitive control) and reading comprehension.

### Demographics and Reading Comprehension

The term of demographics refers to the characteristics of populations including age, SES, IQ, and education. For example, younger children are found having more failures in reading comprehension compared to older children (Jones and Mandeville, 1990). As people age, they have greater difficulty explaining language meanings (Qualls et al., 2001). Moreover, SES plays an important role in children’s literacy development as children from poorer families usually have less literacy experience in contrast with those from rich families (D'angiulli et al., 2004; Myrberg and Rosén, 2008, 2009; Hartas, 2011; Cheng and Wu, 2017). In Aikens and Barbarin (2008), for example, the authors used the Early Childhood Longitudinal Study, Kindergarten Cohort of 1998–1999, to examine the extent to which family, school, and neighborhood factors account for the impact of SES on children’s early reading achievement (measured by Child individual reading assessment). Results found that family characteristics made the largest contribution to the prediction of initial kindergarten reading disparities, which included home literacy environment, parental involvement in school, and parental role strain. The findings imply that multiple contexts are associated with young children’s reading achievement and growth and help account for the robust relation of SES to reading outcomes. In Cheng and Wu (2017), 149 first graders (69 girls and 80 boys) from two urban elementary schools in Shanxi China were recruited to complete SES questionnaire, vocabulary knowledge, morphology awareness, and reading comprehension test. The results observed evidence of associations in SES and reading ability, demonstrating the role of SES in reading comprehension.

The role of IQ, including verbal and performance intelligence, in reading comprehension is largely neglected as more studies focused on influential reading processes. However, some studies have found evidence that IQ (usually measured by Ravens or Wechsler) and reading ability are strongly associated (e.g., Jimenez et al., 2003; Tiu et al., 2003; Ghabanchi and Rastegar, 2014). For example, Tiu et al. (2003) compared reading processes involved in normal children and children with reading disability and found that IQ (measured by Wechsler Intelligence Scale) was an important cause of differences in reading over the processes in the simple view and processing speed. Besides, reading is an important element of education (Thorndike, 1976). Usually, people with longer time of education develop higher ability of reading. Above all, more attention should be paid to the demographics of learners, including age, SES, IQ, and education, in the studies of reading comprehension.

### Language Learning Experience and Reading Comprehension

Studies found that readers with high language proficiency have better reading performance (Golkar and Yamini, 2007; Geva and Farnia, 2012). According to the linguistic threshold hypothesis (Cummins, 1979; Alderson, 1984), after readers reach a certain level of L2 proficiency, L1 reading skills and background knowledge can be transferred to make progress in L2 reading comprehension. In addition, to achieve comprehension in reading, an effective reader should be able to successfully implement strategies such as relating the text with his or her own background knowledge, summarizing information, drawing conclusions, and posing questions at the text.

However, along with strategies, allotting a certain amount of time to reading may be highly effective in increasing reading success. For example, Kirizi (2010) investigated the relationship between reading strategies and time spent on daily reading. The results showed that the use of reading comprehension strategies was a significant predictor on daily time spent reading (*R*^2^=0.57, *p*<0.001), which suggests that there is a significant positive relationship between use of reading strategies and daily time spent on reading. Some studies directly examined the relationship between time spent on reading and reading achievement (e.g., Taylor et al., 1990; Pichette, 2005). For example, Taylor et al. (1990) investigated the effects of time spent on reading at school and at home on intermediate grade students’ reading achievement. One hundred and ninety-five students in Grades 5 and 6 kept daily reading logs from mid-January through mid May. The result of stepwise multiple regression analysis showed that amount of time spent on reading during the reading period contributed significantly to gains in students’ reading achievement. Similarly, Pichette (2005) examined the relationship between time spent on reading and reading comprehension in a second language (L2). Eighty-one French-speaking learners of English (L2), from beginners to advanced, were tested for reading comprehension in French and in English. Low-proficiency learners showed low, non-significant correlations between time spent on reading English and English reading comprehension, but correlations for high-proficiency learners were moderate and significant. The existing literature suggests that language learning experience, particularly language proficiency, and time spent on that language, are significant factors affecting reading comprehension.

### Cognitive Control and Reading Comprehension

Cognitive control is a construct that allocates mental resources and controls behaviors while performing tasks (Miller, 2000; Miyake et al., 2000; Badre, 2008). Cognitive control is not a single cognitive process, but contains multiple components. The most widely used categorization in bilingual research is a framework of Miyake et al. (2000) in which three main components of cognitive control are identified, i.e., inhibition, mental set shifting, and updating and monitoring of working memory. Inhibition concerns one’s ability to deliberately inhibit dominant, automatic, or prepotent responses when necessary. Mental set shifting concerns shifting back and forth between multiple tasks, operations, or mental sets (Monsell, 1996), which involves the disengagement of an irrelevant task set and the subsequent active engagement of a relevant task set. Updating function requires monitoring and coding incoming information for relevance to the task at hand and then appropriately revising the items held in working memory by replacing old, no longer relevant information with newer, more relevant information (Morris and Jones, 1990). Other scholars (e.g., Costa et al., 2009; Green and Abutalebi, 2013; Paap and Greenberg, 2013) have identified conflict monitoring as another independent component of cognitive control, which are generally defined as monitoring one’s performance, internal state, and current environment in response to changing goals.

Reading comprehension is quite cognitive-taxing as individuals have to go through all linguistic levels from phonological level to textual level to understand the meaning of text and form a coherent mental representation of linguistics forms (Kendeou et al., 2014). Some studies have suggested that cognitive control should play a role in reading process (e.g., Kieffer et al., 2013; Fedorenko, 2014; Follmer, 2018; Liu et al., 2018; Cirino et al., 2019), based on behavioral evidence, brain imaging studies, and investigations of brains damage (e.g., Johann et al., 2020; Stasenko et al., 2020). As mentioned above, cognitive control is an umbrella term covering inhibition, shifting and working memory updating and monitoring. Relatively speaking, the role of working memory has been well examined in the reading comprehension studies (e.g., Daneman and Carpenter, 1980; Sesma et al., 2009; Kendeou et al., 2014; Tighe et al., 2015). Referring to other dimensions of cognitive control, some studies demonstrate that shifting supports reading comprehension by allowing readers to flexibly switch from decoding process to meaning, or quickly switch among textual information or series of reading strategies (Kieffer et al., 2013; Cartwright et al., 2017). Besides, inhibition is assumed to be critical in suppressing irrelevant information and inappropriate inferences to facilitate cognitive processes while reading (Cain and Oakhill, 2006; Kieffer et al., 2013). However, as cognitive control is not well defined and has multiple components, how and why a single component is related to reading comprehension remains underdeveloped.

### Chinese (L2) Reading Comprehension

Recent research in this field has advanced understanding in relevant theories and practices, such as reading experiences among heritage speakers (Zhang, 2016), bilingual/multilingual learners (Kim et al., 2016), and processes of reading Chinese (Guan et al., 2011). With more and more university-level students moving to mainland China to learn Chinese, there is a flourish of interest in examining L2 Chinese reading among adults although such studies are relatively few (Zhu, 2021).

Chinese language belongs to Sino-Tibetan language family. Chinese characters are morphologically composed of strokes that form integral characters and compound structures that include radicals and other components. Morphologically, a word is structured by one or more characters that represent meaning units, and there is no space between characters. Syntactically, a simple Chinese clause usually has a basic structure of subject+verb+object (SVO), and relative clauses are usually located before the corresponding nouns. Compared with L1 (e.g., English) reading, L2 Chinese reading has similar reading processes but may entail more effortful cognitive loads (Raudszus et al., 2018), as Chinese language is phonologically, morphologically, and syntactically different from alphabetic languages.

Previous studies in L2 Chinese reading mostly focused on orthography and processing strategies, how L2 readers process information regarding the space and relation between characters and words (e.g., Wang et al., 2003; Lee-Thompson, 2008; Packard, 2008; Shen, 2008), or character/word-level L2 Chinese reading comprehension, sentence/text-level reading comprehension, reading different text genres, sentence structure processing, and reading strategies and skills (e.g., Cao et al., 2013; Cui, 2013; Shen and Jiang, 2013; Xu, 2014; Tong and Yip, 2015; Ke and Koda, 2017; Thoms et al., 2017; Luo and Sun, 2018). Only few studies have explored whether cognitive differences in individuals would explain reading comprehension in Chinese. For example, Chung et al. (2020) investigated the cognitive factors in Chinese adolescent readers with dyslexia and found that both working memory and inhibition played roles in reading comprehension in Chinese and English (L2) after controlling for age and IQ. Liu et al. (2019) took a longitudinal study to explore the bidirectional correlations between cognitive control and word reading abilities in Chinese and English. The results showed that cognitive control was of great importance to Chinese and English reading from kindergarten to early primary school. The results of these studies suggest that reading processes have to include cognitive control to help maintain key information, inhibit irrelevant information, and shift between words and meaning.

Therefore, relatively speaking, few studies have explored L2 Chinese reading, particularly in relation to other variables beyond the Chinese language itself, such as learners’ demographic features, L2 learners’ experience, and cognitive control differences. In this study, we adopt a multiple-perspective view to investigate what factors are significant in predicting L2 Chinese reading success, regarding the variables reviewed above, not only language learning experience, but also learners’ demographic features and cognitive control differences. We expect that all these variables are likely to play significant roles in L2 Chinese reading comprehension. We will examine this issue by investigating Chinese learning students from several different countries by administering a series of questionnaires, tests, and cognitive control tasks.

## Materials and Methods

In the current study, firstly all participants were required to complete comprehensive questionnaires concerning their demographic features (such as IQ and SES), language learning background, and language proficiency. Secondly, all participants were required to complete two cognitive control tasks and a standardized Chinese reading comprehension test. Finally, correlation and multiple stepwise regression analyses were used to explore the relationships between those variables and Chinese reading comprehension. All the final data for statistical analyses are available from https://osf.io/ygv9b/, doi: 10.17605/OSF.IO/UAJCQ.

### Participants

Thirty-four Chinese learners participated in the study. They were international students (10 females, 24 males) from Bangladesh, Burundi, Congo, Madagascar, Malawi, Nigeria, Rwanda, South Africa, and Zimbabwe, who studied mandarin Chinese in Jiangxi Normal University in mainland China. All participants voluntarily participated in the study and gave informed consent, and their rights were protected in accord with the ethical standards of the Academic Committee of Jiangxi Normal University. As all the international students were learning Chinese in China, they not only received the formal instruction of Chinese in classes but also experienced natural Chinese language immersion in different contexts, including negotiating with Chinese teachers, talking with Chinese friends, having Chinese conversation when buying things, etc.

### Materials

In order to collect participants’ demographic background and language proficiency, we adopted the *Language Experience and Proficiency Questionnaire* (Marian et al., 2007), which is widely used in the bilingual research and is significantly correlated with objective measures of language proficiency (Marian et al., 2007; Prior and Gollan, 2011). In the language proficiency questionnaire, native language proficiency was generally rated on a scale from 1 to 10, whereas Chinese (as a foreign language) proficiency was rated separately on listening, speaking, reading, and writing, respectively on a scale from 1 to 10, where 1 corresponded to little skills in that particular language whereas 10 to skills at a native level. The Cronbach’s Alpha of the Chinese proficiency test was 0.940. Moreover, we adopted the Chinese version of *Ravens Matrices* (Raven et al., 1977; Li, 1989) for the measurement of IQ, which is a widely recognized non-verbal test used to measure reasoning and fluid intelligence and is applicable to participants from all cultures, with the Cronbach’s Alpha of reliability analysis=0. 949. For participants’ *SES*, we followed previous literature (Wermelinger et al., 2017; Xie and Zhou, 2020) by adopting their parental education (1–7) as an approximate indicator. Finally, we used the reading comprehension section from *HSK* – *Hanyu Shuiping Kaoshi* level 4 sample tests (we used level 4 because our participants reported to have approximately intermediate level prior to the test) as the *Chinese Reading Comprehension Test*.^1^ HSK is China’s national standardized test designed to assess the Chinese language proficiency of non-native speakers such as foreign students and overseas Chinese (Teng, 2017; Peng et al., 2021). The reading comprehension test comprised sentence completion, sentence ordering, and sentence comprehension, all of which were selective response tasks. There were altogether 40 questions, and thus 40 points for all correct answers (one point for each). The reliability of the Chinese reading comprehension was 0.954 (Cronbach’s Alpha). In the test, participants were required to finish all the questions within 40min. Finally, for the *Cognitive Control* test, we used two commonly-applied tasks, the Flanker task and the Wisconsin Card Sorting Test (WCST), to measure inhibition, monitoring, and shifting of cognitive control, with Cronbach’s Alpha=0.963 for the Flanker task, and Cronbach’s Alpha =0.930 for the WCST.

### Flanker Task

The Flanker task is widely-used to measure inhibition of cognitive control (Eriksen and Eriksen, 1974). Recently it has been also used to reflect monitoring (Costa et al., 2009; Paap and Greenberg, 2013; Xie and Dong, 2017). The Flanker task has a three-condition design: congruent (in which the target arrow is flanked by arrows pointing to the same direction), incongruent (in which the target arrow is flanked by arrows pointing to opposite direction), and neutral (in which the target arrow is flanked by diamonds symbols with no shape similarity to the target).

The Flanker task, designed by E-prime 2.0 following previous literature (Luk et al., 2010; Dong and Xie, 2014; Xie and Dong, 2017), was composed of a practice block with feedback and a formal experimental block without feedback. In the practice block, participants were required to perform with an accuracy rate above 80%, which was to ensure that their attention was focused on the task. Then they would enter into the formal experimental block (altogether 108 trials), with the three conditions evenly distributed. For each trial, a fixation stimulus of “+” appeared for 250ms. Then, a condition of stimulus (congruent, neutral, or incongruent) was presented randomly for 2,000ms, during which participants were required to judge the direction of each target (arrow) by pressing corresponding buttons on the computer keyboard as fast as possible (*F* for <, J for >). This task was completed on computer in a quiet laboratory room.

In the Flanker task, inhibition was measured by calculating the RT differences between incongruent trials and congruent trials. Inhibition reflects the ability to suppress responses that are inappropriate in a given situation (Miyake et al., 2000). Conflict monitoring was measured by the RTs of each condition in the task. Conflict monitoring indicates the ability to monitor one’s performance or internal state or monitor the context and evaluate whether conflict resolution processes should be involved when the target information is presented (Costa et al., 2009; Paap and Greenberg, 2013), the ability to manage attention to a complex set of rapidly changing task demands (Bialystok et al., 2004), or the ability to handle tasks that involve mixing trials of different type that require implementing conflict resolution and those that are free of conflict (Costa et al., 2009).

### The WCST

Wisconsin Card Sorting Test is a widely-recognized task for measuring shifting (Barceló and Knight, 2002; Yudes et al., 2011; Xie and Dong, 2017), although multiple aspects of cognitive control are needed when performing the task. In this test, participants are required to categorize the response card (a combination of numbers, colors, and shapes) according to four stimulus cards (one red triangle, two green stars, three yellow crosses, and four blue circles). With participants sorting the response card, feedback will be given accordingly. If correctness is presented, it means the participant has sorted correctly. Otherwise, the participant has to figure out a different implied rule. Therefore, the participants are continuously sorting the response card changeably in accordance with the feedback. Crucially, the implied rule, which is unknown to the participants, will change after a few trials (5–9 trials).

In the computerized version of WCST programmed in E-prime 2.0 following previous design (Yudes et al., 2011; Xie and Dong, 2017), there were 12 practice trials and 128 formal experimental trials. In each trial, a fixation “+” was presented for 1,000ms. Then, the four stimulus cards appeared in the upper half of the screen while a response card appeared in the lower half of the screen. The participants were required to sort out the response card by pressing designated keyboard buttons (DFJK, each corresponding to a category of stimulus card), followed by a feedback for 1,000ms before the next trial. The participants were told to sort the response card according to the implied rule. All participants were required to complete the 128 trials, with an optional break in the middle. This computerized task was completed in a quiet laboratory room.

In the WCST, the overall response times, the completed categories, the overall errors, and different types of errors were measured. Response times reflect the speed of executing the task, indicating monitoring process. Each completed category means that participant responds correctly to at least five consecutive trials. The overall errors reflect all the errors a participant makes in the task. Perseverative errors indicate that participant fails to change to a new rule when receiving negative feedback. Previous category errors indicate that participant continues sorting the response card according to the previous category despite the negative feedback.

## Results

The results of our study are reported as followed: (1) the descriptive data of all participants’ background information including demographic, linguistic features, and Chinese reading comprehension; (2) the descriptive data of all participants’ cognitive control performance; (3) correlation and multiple regression analyses with L2 Chinese reading comprehension as dependent variable and participants’ demographics (e.g., age, IQ, and SES), L2 learning, and cognitive control as independent variables.

### Demographic and Linguistic Features

All the background information is listed in Table 1. The data showed that on average all participants (*N*=34) were aged 22.79years old (*SD*=1.72). Their SES was 3.15 (*SD*=0.66), and their intelligence (IQ) was 55.91 (*SD*=9.61). By the time of being tested, they had received education for 17.62years (*SD*=2.09), and they had learned Chinese as a foreign language for 3.41years (*SD*=1.80). Their self-rated L1 proficiency was 9.15 (*SD*=1.18), and L2 proficiency was rated as: listening 6.76 (*SD*=1.92), speaking 4.74 (*SD*=2.08), reading 4.82 (*SD*=2.60), and writing 4.79 (*SD*=2.51). We can see that the participants were unbalanced in their L1 and L2 proficiency. Crucially, participants’ Chinese reading comprehension score was 24.50 on average.

**Table 1:** Descriptive data of participants’ background information.

	*N*	Minimum	Maximum	Mean	*SD*
Age	34	20.00	27.00	22.79	1.72
Education (years)	34	15.00	23.00	17.62	2.09
SES (1–7)	34	2.00	5.00	3.15	0.66
IQ (0–72)	34	26.00	70.00	55.91	9.61
L1 Proficiency (1–10)	34	5.00	10.00	9.15	1.18
L2-Chinese Learning History (years)	34	1.00	7.00	3.41	1.80
L2 Listening Proficiency (1–10)	34	2.00	8.00	6.76	1.92
L2 Speaking Proficiency (1–10)	34	1.00	9.00	4.74	2.08
L2 Reading Proficiency (1–10)	34	1.00	9.00	4.82	2.60
L2 Writing Proficiency (1–10)	34	1.00	8.00	4.79	2.51
L2 Reading Comprehension (1–40)	34	4.00	40.00	24.50	11.93

### Cognitive Control Tasks

Table 2 shows the descriptive data of the participants’ cognitive control performance. In the Flanker task, participants responded most quickly in the congruent condition (584ms, *SD*=122.52), moderate in the neutral condition (599ms, *SD*=111.97), and slowest in the incongruent condition (634ms, *SD*=120.35), and the flanker effect (indicating inhibition) was 50ms (*SD*=62.76). Repeated measures analyses showed the effect of conditions within subjects was significant, *F* (2, 66)=15.120, *p*<0.001, *η*^2^ (effect size)=0.314. In the WCST, one participant dropped out of the test because of operation failure. On average, participants completed each trial at 1,873ms (*SD*=565) and achieved correct categories at 5.21 (*SD*=2.62). The overall errors for participants on average were 65.64 (*SD*=15.16), the perseverative errors were 44.70 (*SD*=16.08), and the previous category errors were 35.76 (*SD*=13.08).

**Table 2:** Descriptive data of cognitive control among participants.

	*N*	Minimum	Maximum	Mean	SD
**Flanker Task (RTs)**
Congruent	34	427	877	584	122.52
Neutral	34	436	943	599	111.97
Incongruent	34	455	1,062	634	120.35
Inhibition	34	−128	244	50	62.75
**WCST**
RTs	33	997	3,720	1,873	565
Completed categories	33	1.00	12.00	5.21	2.62
Overall errors	33	36.00	98.00	65.64	15.16
Perseverative errors	33	14.00	75.00	44.70	16.08
Previous category errors	33	12.00	62.00	35.76	13.08

### Correlation Analyses

Correlation analyses were conducted to explore potential relations between participants’ demographic background, language learning, cognitive control, and L2 reading comprehension. Firstly, the result of correlations between demographic variables and L2 reading comprehension (Table 3) showed that demographic factors, such as *age*, *SES*, and *IQ* were not correlated with L2 Chinese reading comprehension (*p*s>0.05). However, education was positively correlated with reading comprehension (*R*=0.409, *p*=0.016). Secondly, correlation analyses between language learning and L2 Chinese reading comprehension (Table 4) showed that only participants’ native language was not correlated with L2 Chinese reading comprehension (*p*=0.480), whereas Chinese learning years, Chinese listening, speaking, reading, and writing proficiency were positively correlated with Chinese reading comprehension (*p*s<0.001). Thirdly, correlations analyses between cognitive control and L2 Chinese reading comprehension (Table 5) showed that only one indicator of the WCST – previous category errors – was negatively correlated with L2 Chinese reading comprehension (*R*=−0.455, *p*=0.008), which means that the more errors participants make, the worse they perform in the Chinese reading comprehension.

**Table 3:** Pearson Correlations between demographic variables and L2 Chinese reading comprehension.

	–	2	3	4	5
1. Age	–	0.441^*^	−0.026	−0.245	−0.078
2. Education		–	−0.178	−0.058	**0.409** ^*^
3. SES			−	−0.262	−0.025
4. IQ				–	0.260
5. L2 Chinese Reading Comprehension					–

* *Correlation is significant at the 0.05 level (two-tailed). The bold indicates the analysis focus*.

**Table 4:** Pearson Correlations between language background and L2 Chinese reading comprehension.

	–	2	3	4	5	6	7
1. L1 Language Proficiency (1–10)	–	0.113	−0.104	−0.058	−0.149	−0.041	−0.125
2. L2 Learning History (years)		–	0.663^**^	0.759^**^	0.677^**^	0.701^**^	**0.812** ^**^
3. L2 Chinese Listening Proficiency (1–10)			–	0.855^**^	0.749^**^	0.731^**^	**0.676** ^**^
4. L2 Chinese Speaking Proficiency (1–10)				–	0.821^**^	0.803^**^	**0.766** ^**^
5. L2 Chinese Reading Proficiency (1–10)					–	0.938^**^	**0.718** ^**^
6. L2 Chinese Writing Proficiency (1–10)						–	**0.698** ^**^
7. L2 Chinese Reading Comprehension							–

** *Correlation is significant at the 0.01 level (two-tailed). The bold indicates the analysis focus*.

**Table 5:** Pearson Correlations between cognitive control and L2 Chinese reading comprehension.

	–	2	3	4	5	6	7	8	9	10
1. Flanker Congruent	–	0.897^**^	0.867^**^	−0.290	0.492^**^	−0.199	0.285	0.303	0.286	−0.281
2. Flanker Neutral		–	0.950^**^	0.105	0.458^**^	−0.123	0.254	0.285	0.166	−0.279
3. Flanker Incongruent			–	0.226	0.398^*^	−0.242	0.354^*^	0.393^*^	0.253	−0.325
4. Flanker Inhibition				–	−0.137	−0.113	0.177	0.220	−0.036	−0.074
5. WCST RTs					–	−0.103	0.154	0.125	−0.060	0.082
6. Overall Errors						–	−0.812^**^	0.973^**^	0.702^**^	−0.270
7. Completed Categories							–	−0.744^**^	−0.523^**^	0.190
8. Perseverative Errors								–	0.770^**^	−0.292
9. Previous Category Errors									–	**−0.455** ^**^
10. L2 Chinese Reading Comprehension										–

* *Correlation is significant at the 0.05 level (two-tailed)*.

** *Correlation is significant at the 0.01 level (two-tailed). The bold indicates the analysis focus*.

### Multiple Regression Analyses

In order to explore what factors might significantly predict L2 Chinese reading comprehension; we conducted stepwise multiple regressions by entering all relevant variables, with the Chinese reading comprehension as dependent variable, with all the demographics, linguistic background, and cognitive control performance as independent variables.

The fittest model of multiple stepwise regressions showed that L2 Chinese learning history, IQ, and previous category errors were the most significant predictors of L2 Chinese reading comprehension, *R*=0.891, adjusted *R*^2^=0.773, *F* (3, 29)=37.417, *p*<0.001, with other variables excluded. According to the model (Table 6), Chinese learning history (*B*=5.220, *t*=8.939, *p*<0.001), IQ (*B*=0.039, *t*=2.857, *p*=0.007), and WCST previous category errors (*B*=−0.207, *t*=−2.529, *p*=0.017) contribute significantly to the result of Chinese reading comprehension.

**Table 6:** Results of multiple regression analyses on Chinese reading comprehension.

Model	*R*	*R* Square	Adjusted *R* Square	*F*	Sig.
Regression	0.891	0.795	0.773	37.417	.000^a^
**Coefficients**	**Unstandardized**		**Standardized**			**B**	**SE**	**B**	**t**	**Sig.**
L2 Learning History	5.220	0.584	0.775	8.939	0.000
Intelligence (IQ)	0.309	0.108	0.253	2.875	0.007
Previous category errors	−0.207	0.082	−0.227	−2.529	0.017

a *Predictors: (Constant), L2 Chinese learning history, IQ, and WCST previous category errors*.

To sum up, the correlation and regression analyses showed that *education, L2 learning history*, and *L2 proficiency* were positively correlated with L2 Chinese reading comprehension, whereas *errors made in the WCST* was negatively correlated with L2 Chinese reading comprehension. Further multiple stepwise regression analyses revealed that *L2 learning history, IQ, and WCST previous category errors* significantly predicted Chinese reading comprehension. These results indicate that longer time of Chinese learning, higher intelligence, and better mental set shifting are related to better performance of Chinese reading comprehension.

## Discussion

The current study examined how demographics, language learning experience, and cognitive control were related to L2 Chinese reading comprehension among international students who learned Chinese as second/foreign language in mainland China. The results showed that demographic features, language learning experience, and cognitive control were indeed related to L2 Chinese reading comprehension, but in different ways.

Firstly, the relationship between demographic features and Chinese reading comprehension seems to be complicated. The correlation results showed that education was positively correlated with Chinese reading comprehension. In the regression analyses when other variables were entered, only IQ remained a significant predictor for variances of Chinese reading comprehension. These results are consistent with previous finding that reading comprehension is associated closely with learners’ demographic features, such as age, IQ, SES, and education (Jones and Mandeville, 1990; Zelinski and Hyde, 1996; Ghabanchi and Rastegar, 2014; Cheng and Wu, 2017). However, we did not observe any significant association between SES, age, and reading comprehension in our study. The reason may lie in some differences between previous studies and ours. Firstly, most previous studies showed that the effect of SES (measured by parental education) on L1 (Chinese) reading achievement has been substantial among children/adolescents (e.g., Guo et al., 2018). As for children, parents with higher SES usually make greater material and interpersonal investments in children’s development (Conger and Donnellan, 2007; Bergen et al., 2017), which may result in larger vocabulary size and more reading activities. However, in our study the participants who took Chinese as second/foreign language were young adults in their early twenties. This may explain why there is no SES effect in our research, which is consistent with some studies that SES is not a significant predictor of L2 (English) reading comprehension among undergraduate students (Ismail et al., 2019). Secondly, different cultures may modify the effect of SES on reading comprehension. It has been reported that Chinese parents tend to have more involvement in their children’s academic learning compared to parents from other cultures as Chinese parents see education as a collective, not an individual responsibility (Huntsinger and Jose, 2009). In our study, however, the participants came from African countries. As for the age effect on reading comprehension, among our participants, their ages were relatively homogeneous as all of them were in their early twenties. This similarity might have reduced the influence of age on reading comprehension.

Our study showed positive relation between IQ and reading comprehension, which is consistent with previous research (Eaves et al., 1994; Tiu et al., 2003; Ningrum and Wibowo, 2017; Blankenship et al., 2019), particularly among children with intellectual disability (Cohen et al., 2001; Tiu et al., 2003). In Blankenship et al. (2019), the results showed that verbal IQ at 6-year-old children had a direct effect on reading achievement. A study of Tiu et al. (2003) tested the role of IQ in a model of reading. A group of children with and a group without reading disability were compared on tests of reading comprehension, listening comprehension, decoding, processing speed, and intelligence (a performance Wechsler Intelligence Scale for Children). The data analysis results showed a significant role of IQ in predicting reading comprehension. However, there are also studies that did not find such association. For example, Share et al. (1989) investigated IQ (Wechsler Intelligence Test) and reading progress in a longitudinal study of an unselected cohort of 741 children whose reading achievement was assessed at ages 7, 9, 11, and 13years. Their findings on rates of progress and levels of achievement clearly indicated that IQ did not set a limit on reading progress, even in extreme low IQ children. Therefore, they did not support the use of IQ tests to determine achievement potential in reading. These results indicate that there are significant correlations between IQ (both verbal and performance intelligence) and L2 reading comprehension. However, the inconsistency suggests that further study should be conducted to verify at what stage or in what occasion IQ matters for reading comprehension. One thing to note is that our study used Ravens Intelligence Test whereas other studies adopted different tests, such as Wechsler Intelligence Test or verbal IQ Test, which further confirmed that the role of IQ in reading comprehension is reliable, although subtle differences may exist between L1 and L2 Chinese reading comprehension. Another thing to note is that IQ was significant in the regression model but not in the correlation analysis. The reason is that when we conducted correlation analyses between demographics and Chinese reading comprehension, we did not enter the variables of language learning (i.e., L1, L2 proficiency, and L2 learning history) and cognitive control performance (the Flanker task and WCST), because we wanted to see the correlations between each aspect of demographics and reading comprehension separately. In regression analyses, however, our purpose was to identify how different variables might work together to predict reading comprehension.

Secondly, the relationship between language learning experience and reading comprehension has been confirmed in the current study. The correlation results showed a positive correlation between L2 proficiency (in listening, speaking, reading, and writing) and L2 reading comprehension. Some studies (e.g., Lee and Schallert, 1997; Jeon and Yamashita, 2014) have reported complex relationship between L2 reading comprehension, L1 reading ability, and L2 proficiency. For example, a study of Lee and Schallert (1997) has shown that the contribution of L2 proficiency is greater than the contribution of L1 reading ability in predicting L2 reading ability, and that a threshold level of language proficiency (Clarke, 1980) exists such that learners with low levels of L2 proficiency will show little relationship between their L1 and L2 reading ability whereas learners with higher levels of L2 proficiency will show a positive relationship between their L1 and L2 reading performance. In our study, there was no relationship between L1 proficiency and L2 reading comprehension, possibly because our participants’ L2 (Chinese) proficiency was relatively low.

L2 learning history was significantly related with L2 reading comprehension in both correlation and multiple regression analyses. Previous studies did not directly define and examine the effect of L2 learning history on reading comprehension, but examined how time spent on reading might affect reading comprehension. For example, reading volume (time spent on reading) has been suggested as one of the key factors improving reading achievement (Allington, 2014). Likewise, in a large-scale observational study, Foorman et al. (2006) found that the key factor of the reading instruction offered by over 100 observed 1st and 2nd grade teachers was the time that they allocated to text reading. In their findings, reading volume during reading explained variance observed on any of the outcome measures, including word recognition, decoding, and reading comprehension, whereas other time factors, including time spent on phonemic awareness, word recognition, or decoding, were not related to reading growth. These similar results, including ours, clearly show that time spent on L2 learning, or L2 learning history (as broadly termed in our study) has a significant relationship with L2 reading comprehension.

Thirdly, our study provides evidence that cognitive control contributes to L2 reading comprehension. Both the correlations and the regressions analyses in our study showed that the previous category errors made in the WCST were significantly related with L2 reading comprehension. These results are consistent with recent findings that L2 reading comprehension is related with cognitive control (Zirnstein et al., 2018; Meixner et al., 2019; Chung et al., 2020; Johann et al., 2020; Wu et al., 2020). Most previous studies have reported that working memory contributes substantially to L2 reading comprehension (Linck et al., 2014; Nouwens et al., 2016; Weng et al., 2016; Jung, 2018; Johann et al., 2020; Hijikata and Koizumi, 2021). However, relatively fewer studies examined other components of cognitive control and their relationship with L2 reading. In our study, we adopted the Flanker task and the WCST to measure three components of cognitive control: inhibition, monitoring, and mental set shifting. We did not observe the significant effect of inhibition and monitoring as measured in the Flanker task. The study of the relationship between the component of monitoring and L2 reading is relatively new, and we have not seen similar studies in the literature. However, there are a few studies examining inhibition and reading comprehension. For example, in Chung et al. (2020), which examined participants with dyslexia, it was found that working memory, inhibition, shifting, vocabulary knowledge, and rapid naming contributed uniquely to reading comprehension in L2 (English). However, our study did not find the inhibition effect, and the participants were college students and they took Chinese as second/foreign language in a naturalistic Chinese immersion context, which may exert differential impact on cognitive control.

In our study, we found that mental set shifting as measured in the WCST significantly contributed to L2 Chinese reading comprehension, which is at least partially consistent with some studies in the literature. For example, in Johann et al. (2020), 186 school children were examined by administering a complex span task (working memory), task switching (cognitive flexibility), a Stroop-like task (inhibition), raven matrices (IQ), a reading speed task, and three reading comprehension tasks. The results showed that working memory, inhibition, and IQ were related to reading speed, but cognitive flexibility (mental set shifting) and IQ were related to reading comprehension, which is similar to our finding. The difference is that we used the Flanker task for inhibition and the WCST for mental set shifting. As mentioned in the introduction, the reason why mental set shifting plays a role in reading comprehension is that shifting ability allows readers to flexibly switch from one mental set to another, such as shifting from decoding process to meaning, or quickly switching among textual information or series of reading strategies (Kieffer et al., 2013; Cartwright et al., 2017). Furthermore, as our participants were in a naturalistic Chinese immersion context, it could possibly be easier for them to switch between languages, thus increasing the efficiency of mental set shifting (Xie and Antolovic, 2021). All these findings suggest that the component of mental set shifting is reliably related to L2 reading comprehension. Combined with findings from other studies, it suggests that different component of cognitive control may contribute to reading comprehension distinctively, which deserves further verification in future studies.

## Limitations and Future Concerns

The current study has provided evidence of the differential contributions of demographics, L2 learning history, and cognitive control to L2 (Chinese) reading comprehension. However, there are some limitations, which are also insightful for future concerns. Firstly, the sample size in our study was relatively small. We believe that a large-scale sample will certainly bring us a more comprehensive picture of the potential variables affecting reading comprehension. Secondly, our study did not include L1 Chinese readers for investigation, but future studies should compare differences between CFL learners and Chinese native speakers as Chinese language may recruit differential cognitive resources compared to alphabetic languages. Previous studies show that visual-verbal association skill significantly predicts Chinese reading acquisition among Chinese native speakers, as the Chinese writing system is not phonologically based but heavily visual (see Yang et al., 2013 for meta-analysis). Moreover, this visual strategy in Chinese reading may be transferred to L2 (English) reading (Koda, 1998; Jeon and Yamashita, 2014). Thirdly, the relationship between those factors examined and Chinese reading comprehension is not clarified regarding the causality. Future studies are encouraged to conduct experimental or longitudinal research for this purpose.

## Conclusion

The current study explored whether demographic factors, L2 learning experience, and cognitive control contributed to the L2 Chinese reading comprehension. Both the correlation and the stepwise multiple regression analyses showed that some aspects of the three variables significantly contributed to L2 Chinese reading comprehension, such as education and IQ of demographics, L2 proficiency and history of L2 learning experience, and mental set shifting of cognitive control. These findings suggest that L2 reading competence is related not only to language learning itself but also to learners’ demographic background and individual differences of cognitive control, which also provide implications for both L1 and L2 language learning. Future studies should further clarify when and why only some aspects of those variables matter under a specific context.

## Data Availability Statement

The datasets presented in this study can be found in online repositories. The names of the repository/repositories and accession number(s) can be found at: https://osf.io/ygv9b/, doi: 10.17605/OSF.IO/UAJCQ.

## Ethics Statement

The studies involving human participants were reviewed and approved by the Academic Committee of Jiangxi Normal University. The patients/participants provided their written informed consent to participate in this study.

## Author Contributions

ZX designed the research and drafted the manuscript. QQ coordinated and organized the data collection process. WW, XC, QQ, FY, JH, MC, and ZX collected the data and made the data analyses. All authors contributed to the article and approved the submitted version.

## Funding

This research received grants from the National Social Science Fund of China (19BYY083).

## Conflict of Interest

The authors declare that the research was conducted in the absence of any commercial or financial relationships that could be construed as a potential conflict of interest.

## Publisher’s Note

All claims expressed in this article are solely those of the authors and do not necessarily represent those of their affiliated organizations, or those of the publisher, the editors and the reviewers. Any product that may be evaluated in this article, or claim that may be made by its manufacturer, is not guaranteed or endorsed by the publisher.

## Footnotes

^1^ http://www.chinesetest.cn
